# The role of macrophage migration inhibitory factor in ocular surface disease pathogenesis after chemical burn in the murine eye

**Published:** 2010-11-17

**Authors:** Sei Yeul Oh, Jong-Sun Choi, Eo-Jin Kim, Roy S. Chuck, Choul Yong Park

**Affiliations:** 1 Department of Ophthalmology, Sungkyunkwan University School of Medicine, Samsung Medical Center, Seoul, South Korea; 2 Department of Pathology, Dongguk University Seoul, Graduate School of Medicine, Seoul, South Korea; 3 Department of Ophthalmology and Visual Sciences, Montefiore Medical Center and Albert Einstein College of Medicine, New York, NY; 4 Department of Ophthalmology, Dongguk University Seoul, Graduate School of Medicine, Seoul, South Korea

## Abstract

**Purpose:**

To evaluate the role of macrophage migration inhibitory factor (MIF) in the wound healing process following severe chemical burns to the ocular surface.

**Methods:**

Chemical burning of the ocular surface was induced in mice (C57BL/6) via the application of 0.1 M NaOH. Macrophage migration inhibitory factor (*MIF*), tumor necrosis factor-α (*TNF-α*), and interleukin-1β (*IL-1β*) mRNA expression in the ocular surface and lacrimal gland was evaluated via real-time reverse transcription PCR on days 2, 7, and 30 after induction of the chemical burn. The expression of MIF protein in the ocular surface and lacrimal gland was evaluated via western blot analysis. Immunohistochemical staining was conducted to detect MIF and vasculoendothelial growth factor in the cornea during the wound healing process. The angiogenic role of MIF was further evaluated using an 8–0 polyglactin suture technique to induce corneal neovascularization.

**Results:**

*MIF*, *TNF-α*, and *IL-1β* mRNA expression were elevated significantly in the ocular surface up to day 30 after chemical burn induction. *TNF-α* alone was elevated in the lacrimal gland. MIF protein elevation was confirmed via western blot analysis, and the spatial similarity of MIF and VEGF expression in the cornea was noted during the wound healing process. 8–0 polyglactin sutures soaked in MIF induced significantly higher numbers of new vessels on the mouse cornea after 7 days (p=0.003, Mann–Whitney test).

**Conclusions:**

These findings indicate that MIF performs a crucial role in wound healing on the ocular surface after the induction of chemical burns.

## Introduction

Macrophage migration inhibitory factor (MIF) was initially described as a soluble factor identified during the delayed hypersensitivity reaction [1]. The presence of macrophages at the inflamed site is the origin of the nomenclature of this interesting cytokine [1,2]. Therefore, the role of MIF was initially recognized as a unique pro-inflammatory cytokine and was considered essential for T-cell activation. However, the results of recent studies have revealed that the role of MIF is not limited to inflammation; rather, it is also profoundly involved in the regulation of cellular growth [2], wound healing [3], tissue development, and even tumor formation under a variety of pathologic conditions [4]. Previous investigations have revealed MIF expression in a variety of tissues, including the brain, ear, lung, breast, liver, testis, vascular endothelial cell, vascular smooth muscle cells, skin, kidney, endocrine glands (pituitary gland, adrenal cortex, and pancreas), and eye (lens, cornea, iris, ciliary body, lacrimal gland, and retina) [5,6].

The vital function of MIF in the wound healing process was studied previously. Elevated local levels of MIF were associated with elevated inflammatory response and marked delays in wound healing in human and mouse skin wound models [3]. On the other hand, other studies have demonstrated that MIF promotes fibroblast migration and accelerates skin wound healing [7,8]. A close relationship between MIF and vascular endothelial growth factor (VEGF) expression has recently been reported [9,10].

The wound healing response after tissue injury is critical to the individuals’ survival. In nonocular tissues, the cicatrical change accompanying new vessels and strong fibrosis may be beneficial, providing superior structural stability. However, considering the transparent nature of the cornea and the smooth and wet conjunctival surface of the normal ocular environment, it is clearly necessary to minimize fibrosis and new vessels in the process of wound healing following ocular surface trauma. One good example of this is chemical burns to the ocular surface, especially those induced by alkaline agents. This condition characterized by severe inflammation disrupts or destroys the normal homeostasis of corneal and conjunctival epithelial cells [11]. The induced deficiency of the limbal stem cell population of corneal epithelium results in clinically significant opacity, new vessel formation, and persistent epithelial corneal defects [11-13]. The cicatrical change in the conjunctiva results in tear film abnormalities and dry eye [11-13].

During the wound healing process, many cytokines secreted either from ocular resident cells or from recruited inflammatory cells are involved in the pathogenesis. Tumor necrosis factor- α (TNF- α), interleukin-1 (IL-1), interleukin-6, interleukin-10, and VEGF are some of the cytokines that were previously studied extensively in ocular chemical burn experiments [14-18]. And MIF is also a strong candidate molecule. Because MIF is also closely related to both fibrosis and neovascularization [7-10], the role of MIF in ocular wound healing was previously studied. MIF expression in the cornea was upregulated in animal models after mechanical corneal damage or infection [19,20]. MIF knockout mice exhibited reduced corneal neovascularization relative to the wild-type mice following corneal nylon suture or alkali burn [19]. These studies implied that MIF might perform an important function in ocular cicatrical changes occurring after trauma.

Recently our group reported a high level of MIF expression in the normal murine lacrimal gland and a dynamic change of MIF expression in an experimentally induced murine dry eye model [6,21,22]. In previous studies we induced local change in the lacrimal gland via a focal injection of botulinum toxin type B into the main lacrimal gland. This induced experimental dry eye and was associated with the sustained elevation of MIF expression within the lacrimal glands. However, no significant changes in MIF in the ocular surface were detected [21,22]. In a clinical setting, dry eye and chemical burn differ profoundly in pathogenesis. Chemical burns destroy the ocular surface far beyond the extent of dry eye. Although no significant role for MIF on the ocular surface was detected in our dry eye model, the role of MIF in chemical burns could differ profoundly.

In the present study we induced severe inflammation on the ocular surface via the induction of chemical burns (NaOH) in mice. We explored the expression of MIF, TNF-α, and IL-1 both in the ocular surface and lacrimal gland via real-time reverse transcription PCR (real-time RT–PCR) and western blot analysis. Vascularization of the cornea is the ultimate result of severe ocular surface inflammation, and VEGF is considered a major regulator of new corneal vessels. Thus, we evaluated the possible relationship existing between VEGF and MIF expression in corneal cells during the process of wound healing after the induction of chemical burns via immunohistochemistry. Furthermore, we evaluated the effects of exogenous MIF on induced corneal neovascularization using a suture-induced model.

## Methods

### Animals

Ninety-three male, 7-week-old C57BL/6 mice (Dooyeol Biotech, Seoul, Korea) were used in accordance with the Association for Research in Vision and Ophthalmology Statement for the Use of Animals in Ophthalmic and Vision Research. The experimental protocol was approved by the Institutional Animal Care and Use Committee of Dongguk University.

Ten mice were used as a control group. The eyes and lacrimal glands were harvested after euthanizing the animals (overdose sedation using xylazine and ketamine before cervical dislocation). Ten samples from the right eyes were used for RNA extraction and real-time RT–PCR, and 10 samples from the left eyes were fixed in formalin and used for histology and immunohistochemistry. Another 75 mice were divided into groups 1, 2, and 3 (n=25, each group). After induction of chemical burns in the right eyes (0.1 M NaOH, 20 µl application on the ocular surface for 30 s with no washing), group 1 mice were sacrificed 48 h after chemical burn induction, group 2 mice were sacrificed 7 days after chemical burn induction, and group 3 mice were sacrificed 30 days after chemical burn induction. The eyes and lacrimal glands were harvested from both the right and left sides. The tissues from the left side were used as controls. Fifteen right eyes of each group were used for RNA extraction and real-time RT–PCR, five right eyes of each group were used for histology and immunohistochemistry, and five right eyes of each group were used for protein extraction and western blot analysis. Another eight mice received 8–0 polyglactin (Catalog no:W9560; Coated Vicryl; Ethicon, Cornelia, GA) corneal sutures to induce the formation of new corneal vessels.

### Quantitative real-time reverse transcription PCR

Enucleation was performed, and the main lacrimal gland was harvested en bloc. The ocular surface tissues containing the whole cornea, conjunctiva, and the anterior half of the sclera were obtained. Total RNA was isolated from the ocular surface and lacrimal gland using an RNeasy kit (Qiagen, Valencia, CA) in accordance with the manufacturer’s instructions. The RNA concentrations were determined via ultraviolet spectrophotometry. RNA samples were treated with DNase I (Catalog No. 18047–019; Invitrogen, Carlsbad, CA) to preclude genomic DNA contamination. The first-strand cDNA was synthesized from 1 µg of total RNA with oligo d'-T primer using a commercially available kit (SuperScript^TM^ Ш Reverse Transcriptase; Invitrogen, Carlsbad, CA). Aliquots of samples of cDNA were stored at –80 °C until use. Real-time quantitative PCR was conducted and analyzed with a Roche Light-Cycler. (LightCycler 1536; Roche, Basel, Switzerland) Reactions in a 20 µl volume were conducted using SYBR Green reaction mix (Qiagen) with 0.5 mM of primer. Cyclophilin A was used as normalization standard. Assays were conducted in triplicate. The measured value of the right organ was compared to the measured value of the left organ in each mouse. The value of the right organ divided by the value of the left organ was employed for analysis. The sequences of the PCR primer pairs are listed in Table 1.

**Table 1:** Primer sequences for PCR.

**Gene**	**GeneBank acession**	**Left primer**	**Right primer**	**PCR product (bp)**
*IL-1β*	NM_008361	GCCCATCCTCTGTGACTCAT	AGGCCACAGGTATTTTGTCG	229
*TNF- α*	NM_013693	GAACTGGCAGAAGAGGCACT	AGGGTCTGGGCCATAGAACT	201
*MIF*	NM_010798	GTGCCAGAGGGGTTTCTGT	AGGCCACACAGCTTACT	205
Cyclophilin A	XR_004644	CAGACGCCACTGTCGCTTT	TGTCTTTGGAACTTTGTCGCAA	132

### Histology and immunohistochemistry

Harvested tissues were formalin fixed and paraffin blocks were prepared. Four micron-thick sections were obtained from formalin-fixed, paraffin-embedded tissues, transferred onto adhesive slides, and dried at 60 °C for 40 min. The immunohistochemical procedures were then conducted using a BenchMark XT automatic immunohistochemical staining device (Ventana Medical System, Tucson, AZ). After dewaxing and rehydrating, antigen retrieval was performed. The slides were then incubated for 30 min at 42 °C with MIF polyclonal antibodies (1:2,000, Catalog no: 36–7401; Zymed, Carlsbad, CA) or VEGF monoclonal antibodies (1:200, Catalog no: ab1316; Abcam, Cambridge, MA). The primary antibodies were detected using an iVIEW DAB detection kit (Ventana Medical System).

### Western blot analysis

For western blot analysis, the ocular surface tissues stored at –80 °C were quickly homogenized with a tissue crusher while frozen, and the tissue powder was placed into boiling lysis buffer (1% sodium dodecyl sulface, 1.0 mM sodium ortho-vanadate, 10 mM Tris [pH 7.4]), placed into a microwave oven for 15 s, and centrifuged for 5 min at 11,400× g at 15 °C. The samples were then separated via SDS–PAGE under denaturing conditions and electroblotted onto a polyvinylidine difluoride membrane (BioRad, Hercules, CA). After being blocked using 5% nonfat dry milk in Tris-buffered saline containing 10 mM Tris (pH 7.6), 150 mM NaCl, and 0.1% Tween-20, the membranes were incubated with rabbit antimouse MIF polyclonal antibodies (Catalog no: 36–7401; Zymed) and diluted to 1:200 in blocking solution overnight at 4 °C. The membranes were further incubated with an antirabbit horseradish peroxidase-conjugated antibody (Santa Cruz Biotechnology, Santa Cruz, CA). They were then treated with an enhanced chemiluminescence solution (ECL kit; Pierce, Rockford, IL), and the signals were captured on an image reader (Las-3000; Fuji Photo Film, Tokyo, Japan). To monitor the amount of protein loaded into each lane, the membranes were treated with a stripping buffer and reprobed with a monoclonal antibody against β-actin (Sigma-Aldrich, St Louis, MO). The protein bands were analyzed via densitometry. The ratio between the control and sample was calculated asfollows:

$$\text{Ratio}=\frac{\text{right tissue (density of MIF divided by density of }\beta \text{-actin}}{\text{left tissue (density of MIF divided by density of }\beta \text{-actin}}$$

### Mouse corneal neovascularization assay

Mouse corneal neovascularization was induced using a 3-mm bite length of 8–0 polyglactin suture near the limbus. Eight mice were used. The right eyes received sutures incubated in recombinant mouse MIF (10 μg/ml, Catalog No: 1978-MF; R&D Systems, Minneapolis, MN) for 48 h. The left eyes received sutures incubated in PBS (Catalog No: 14190–250, no magnesium, no calcium, no phenol red; GIBCO, Grand island, NY) for 48 h. After 7 days, both the cornea and conjunctiva were digitally photographed (Nikon D3000; Nikon, Tokyo, Japan) and angiogenesis scoring was conducted. Angiogenic activity was scored as the number of newly developed vessels crossing the limbus, which was readily visible on the pictures. Mean angiogenic activity was compared between the right and left eyes.

### Statistical analysis

SPSS version 12.0 for Windows (SPSS Inc. Chicago, IL) was used for statistical analysis. Mann–Whitney tests were conducted for analysis of difference between the two groups. The Kruskal–Wallis test and least significant difference test (using ranks for multiple comparisons) were used for analyses of the difference between three or more groups. A p value of less than 0.05 was prospectively assigned as the threshold for statistical significance.

## Results

### Histology of chemical burn of murine ocular surface

**Figure 1:**
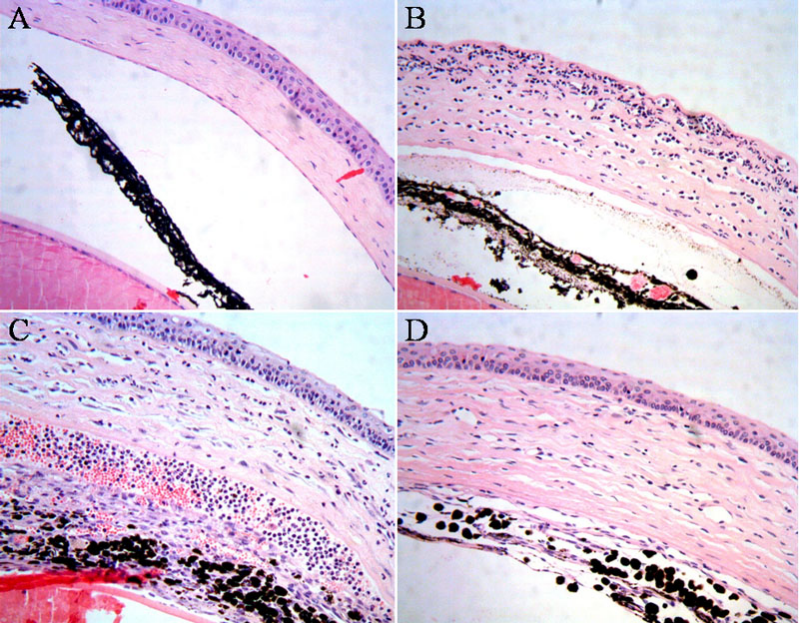
Histology of the mouse eye after induction of chemical burns by 0.1 M NaOH. Chemical burns induced with 0.1 M NaOH induced severe inflammatory changes on the ocular surface. Representative figures are shown. **A**: Control eye, no chemical burn induced. **B**: Two days after chemical burn. Severe infiltration by inflammatory cells and total loss of the epithelial layer are observed. **C**: Seven days after chemical burn. Corneal inflammation is reduced compared to day 2; corneal edema persists; epithelial layer has regenerated. **D**: Thirty days after chemical burn. Inflammation has almost completely subsided; however, marked increases in corneal stromal thickness and cellularity persist.

Eyes evidenced severe inflammation after the induction of chemical burns by NaOH. The corneas were grossly swollen and hazy. Heavy infiltration by inflammatory cells was observed at full thickness in the cornea, and was particularly severe in the superficial layer. Occasionally, the total absence of epithelial layers and a marked reduction in endothelial cells were noted. This acute change recovered partially over 4 weeks, although the increased corneal thickness and cellularity persisted (Figure 1).

### Macrophage migration inhibitory factor and vascular endothelial growth factor immunohistochemistry

**Figure 2:**
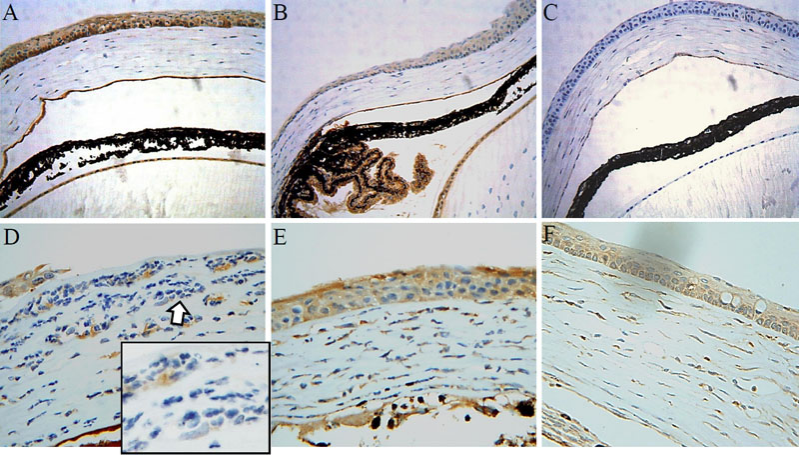
Macrophage migration inhibitory factor immunohistochemistry in normal and chemical burn applied to the mouse eye. In normal control eyes, MIF was expressed in the corneal epithelium, corneal endothelium, lens epithelium, and ciliary body epithelium (**A**, **B**). However, it was difficult to find any significant MIF staining in the corneal stroma. **C**: Negative control; secondary antibody staining without primary antibody application. **D**: MIF expression was detectable in corneal stroma at day 2 after chemical burn induction. However, the infiltrating inflammatory cells were largely negative for MIF expression (inserted figure in panel **D** is the magnification of area indicated by the clear arrow). **E**, **F**: MIF staining on days 7 and 30 after chemical burn. MIF expression in the corneal stroma was increased but cellular infiltration was reduced compared to day 2.

MIF was expressed in the corneal epithelium, corneal endothelium, conjunctival epithelium, lens epithelium, and ciliary body epithelium. In the epithelial layer of the cornea, MIF staining was positive with more prominent expression in the basal cells. The corneal stromal layer was negative for MIF staining (Figure 2).

**Figure 3:**
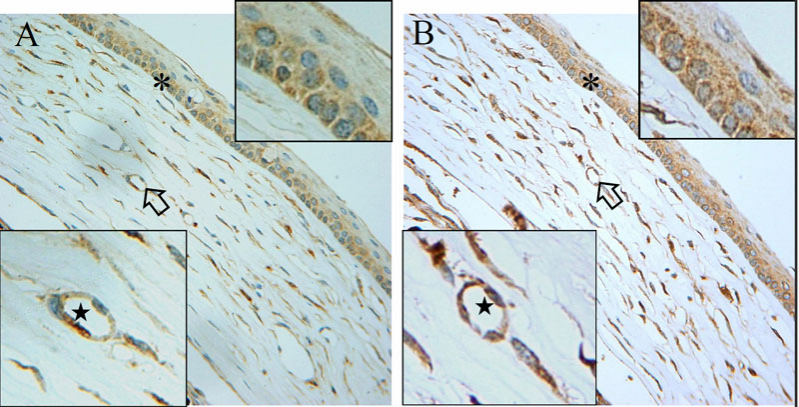
Macrophage migration inhibitory factor and vascular endothelial growth factor immunohistochemistry after chemical burn of ocular surface. Thirty days after induction of chemical burns on the ocular surface, the murine corneas were stained with antibodies against MIF (**A**) and VEGF (**B**). The inflammation almost completely subsided, and irregularly distributed corneal stromal fibers were noted, with a few microvessels (arrow). Both cytokines were readily observable in the corneal stroma and epithelium. Their expression revealed high spatial similarity as demonstrated in the inserted magnified images. In the epithelial cell layer, both MIF and VEGF were heavily expressed in basal cells as compared to cells in the superficial layers. Both VEGF and MIF were positively stained in cells around microvascular structures (asterisk). The inserted magnified image represents the areas indicated by the arrow and asterisk.

After chemical burn induction, MIF staining could be readily observed in the corneal stromal layer in all samples. However, the infiltrating inflammatory cells were mostly negative for MIF. Interestingly, MIF was expressed in cells around the microvessels and evidenced a high degree of spatial similarity with VEGF expression (Figure 3). Whereas the corneal changes were prominent, the conjunctival changes were difficult to discriminate, owing to scanty tissue in the mice.

### Real-time RT–PCR for macrophage migration inhibitory factor, interleukin-1β and tumor necrosis factor-α

**Figure 4:**
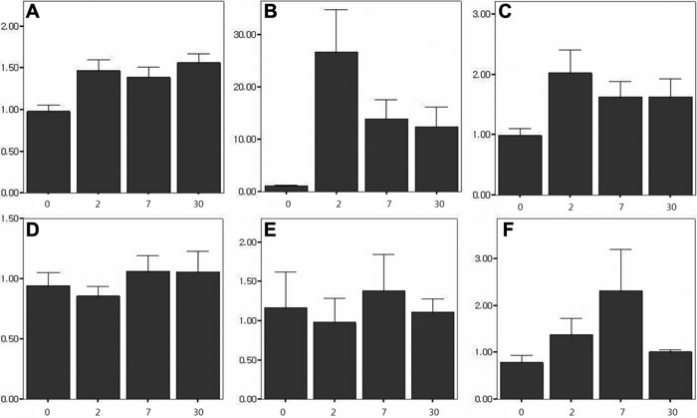
Real-time RT–PCR for macrophage migration inhibitory factor, interleukin-1β, and tumor necrosis factor-α in the ocular surface and lacrimal gland after chemical burn (at days 2, 7, and 30 after injury). **A**-**C**: Ocular surface; panels **D**-**F**: lacrimal gland. **A**: MIF expression. MIF is elevated in the ocular surface after chemical burn induction on days 2, 7, and 30 relative to the controls. P values: day 0 versus day 2 (p<0.001), day 0 versus day 7 (p=0.003), day 0 versus day 30 (p=<0.001), otherwise not significant. **B**: The expression of IL-1β. IL-1β evidenced dramatic changes in the ocular surface after chemical burn induction. P values: day 0 versus day 2 (p<0.001), day 0 versus day 2 (p=0.001), day 0 versus day 3 (p=0.002), otherwise not significant. **C**: TNF-α expression. TNF-α is elevated in the ocular surface after chemical burn. P values: day 0 versus day 2 (p=0.009), day 0 versus day 7 (p=0.032), day 0 versus day 30 (p=0.049), otherwise not significant. **D**: The expression of MIF. **E**: The expression of IL-1β. **F**: The expression of TNF-α. Both MIF and IL-1β evidenced no significant changes in the lacrimal gland after chemical burn. However, TNF-α is elevated in the lacrimal gland after chemical burn. P values: day 0 versus day 2 (p=0.001), day 2 versus day 7 (p=0.020), otherwise not significant. Error bars show mean±1.0 standard error; bars show means.

The mRNA expression levels of *MIF*, *IL-1β*, and *TNF-α* were evaluated via real-time RT–PCR. In the ocular surface, *MIF*, *IL-1β*, and *TNF-α* expression were significantly elevated after chemical burn induction on days 2, 7, and 30. *MIF* and *TNF-α* expression increased steadily for 4 weeks after injury. However, the elevation of *IL-1β* was dramatic at day 2 and then decreased over a 4-week period but consistently was maintained at elevated levels relative to the controls (Figure 4). *MIF* and *IL-1β* in the lacrimal gland evidenced no significant changes after chemical injury to the ocular surface. However, the expression level of *TNF-α* was increased significantly on days 2 and 7 after injury. This change in *TNF-α* normalized on day 30 after injury (Figure 4).

### Western blot analysis

**Figure 5:**
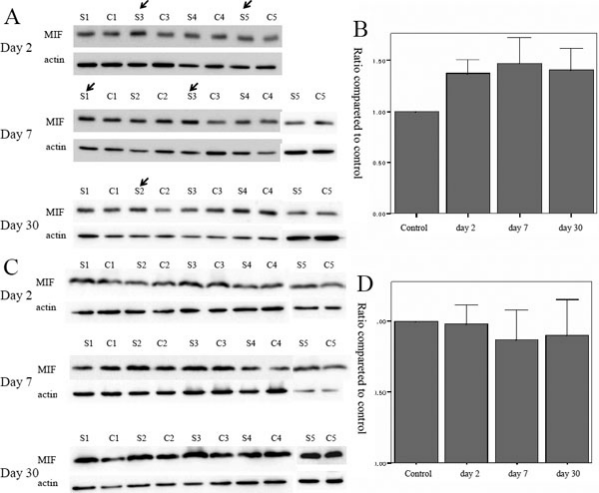
Western blot for macrophage migration inhibitory factor in the ocular surface and lacrimal gland after chemical burn (at days 2, 7, and 30 after injury). **A**: western blot for MIF in the ocular surface. S is the study eye and C is the control eye. The number designates the serial number of each mouse. The arrows indicate prominent elevations of MIF in the study eyes relative to the controls. **B**: Means and standard errors of density calculated from western blotting. MIF is elevated in the ocular surface on days 2, 7, and 30 after chemical burn. P values: control versus day 2 (p=0.004), control versus day 7 (p=0.028), control versus day 30 (p=0.010), otherwise not significant. **C**: western blot for MIF in the lacrimal gland. S is the study eye and C is the control eye. Numbers designate the serial number of each mouse. **D**: Means and standard errors of density calculated from western blot. MIF evidenced no significant differences in the lacrimal gland after chemical burn. Error bars show mean±1.0 standard error; bars show means.

Western blot analysis of MIF in the ocular surface revealed significant elevation on days 2, 7, and 30 (Figure 5). However, western blot analysis of MIF in the lacrimal gland revealed no significant elevation after chemical burn induction (Figure 5).

### Induction of mouse corneal neovascularization with macrophage migration inhibitory factor

**Figure 6:**
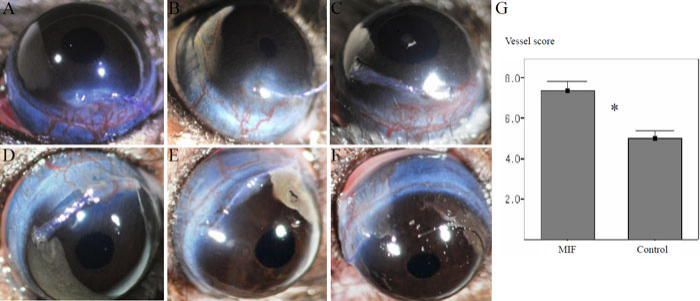
Macrophage migration inhibitory factor enhanced mouse corneal neovascularization after suture. An 8–0 polyglactin suture was placed in the peripheral cornea near the limbus. Seven days later, the corneal neovascularization was assayed by counting new vessels crossing the limbus (vessel score). **A**-**C**: Representative pictures of neovascularization after MIF-incubated polyglactin suture (vessel scores: 8 [**A**], 6 [**B**], and 10 [**C**]). **D-F**: Representative pictures of neovascularization after PBS-incubated polyglactin suture (vessel scores: 6 [**D**], 5 [**E**], and 5 [**F**]). The violet color of the suture is visible on the corneas. **G**: Vessel score of the MIF group is significantly higher than that of the PBS control group (asterisk; p=0.003, Mann–Whitney test). Error bars show mean±1.0 standard error; bars show means.

Sutures induced significant corneal neo-vessels, using both PBS-incubated or MIF-incubated polyglactin. However, angiogenic activity (vessel score) was significantly higher in the MIF-incubated polyglactin (p=0.003, Mann–Whitney test) relative to the PBS controls on day 7 after suture placement (Figure 6).

## Discussion

Our data reveal that MIF plays a prominent role in the alkali-burned murine eye. On the ocular surface after chemical burn induction, MIF protein was expressed at elevated levels (real-time RT–PCR and western blot) and was abundantly noted in the corneal stromal cells, which normally lack MIF expression. The infiltrating inflammatory cells were negative for MIF expression. Additionally, MIF expression was prominent in the cells surrounding microvessels and was co-localized with VEGF expression. Real-time PCR showed that the kinetics of *MIF* expression were similar to other inflammatory cytokines, including *TNF-α* and *IL-1β*. *MIF* mRNA was elevated in the ocular surface for 4 weeks after injury. In the lacrimal glands, MIF protein and mRNA were not elevated at any time. Because VEGF is one of the best known cytokines involved in the induction of new vessels under pathologic conditions, the co-existence of MIF and VEGF in similar locations indicates that MIF plays an important role in corneal neovascularization. Here, we induced new vessels using a suture technique and demonstrated that the exogenous application of MIF together with sutures significantly enhances new vessel formation in the mouse cornea.

MIF performs many interesting biologic functions. MIF has been described previously as important in the immune response [1,2,5], angiogenic [23,24], both a promoter and an inhibitor of wound healing [3,7,8], a promoter of fibrosis [8,25-28], an upregulator of matrix metalloproteinases in the promotion of vessel growth [29-31], and a promoter of angiogenic factors, such as VEGF and IL-8 [32,33].

In our study we determined that MIF is abundantly expressed in corneal stromal cells, and the distribution of MIF in the corneal stroma closely matches that of VEGF. This finding is consistent with previous reports revealing that MIF promotes VEGF and together with the destruction of stroma via the upregulation of metalloproteinases, MIF and VEGF may enhance the formation and growth of new vessels. Additionally, we determined that MIF expression was absent in infiltrating corneal inflammatory cells; this result differs from the previously reported finding that MIF was found principally in infiltrating cells in the corneal stroma after ocular surface injury [19]. The finding that MIF expression in the corneal stroma increased while inflammation subsided during the wound healing process also reflects that increased MIF originated principally from corneal stromal cells.

Interestingly, the cells involved in microvessel construction were positive for both MIF and VEGF. Furthermore, the kinetics of MIF expression were correlated with inflammatory cytokines, such as TNF-α and IL-1. This suggests that MIF interacts not only with VEGF but also with other inflammatory cytokines in our ocular chemical burn model. MIF was previously determined to induce TNF-α and IL-1 β expression from the peripheral blood mononuclear cells [34]. Additionally, TNF-α was reported to induce the secretion of MIF from dendritic cells and ovarian cancer cells [35,36]. Recently, anti-TNF therapy resulted in reduced serum MIF levels in patients with rheumatoid arthritis [37]. Therefore, these cytokines may cross-regulate one another in the pathologic cornea and participate in the wound healing response.

Whether the elevation of MIF on the ocular surface promotes or delays wound healing remains to be clearly elucidated. In a previous report it was determined that MIF was advantageous for corneal recovery after ultraviolet-induced photokeratitis in mice [38]. However, when considering both the pro-inflammatory and angiogenic effects of MIF, further studies will be necessary to clarify the role of MIF in ocular surface wound healing.

It is interesting to note that MIF and IL-1β in the lacrimal gland evidenced no significant changes during the wound healing process after chemical burn induction, whereas TNF-α in the lacrimal gland was significantly elevated in the same period. In our previous reports using the botulinum toxin B-induced murine dry eye model, TNF-α and IL-1β were increased on both the ocular surface and lacrimal gland, whereas MIF was increased only in the lacrimal gland [21,22]. Considering the differences in pathogenesis between chemical burn and dry eye, the discrepancy in cytokine expression in the two different disease models is quite understandable.

In the clinical setting the wound healing response after severe ocular surface chemical burns is usually accompanied by corneal haziness, fibrosis, and neovascularization [11]. A variety of therapeutic strategies, including limbal stem cell transplantation, amniotic membrane application, and autologous serum eye drops, have been used in attempts to re-establish the destroyed ocular surface following severe chemical burns [11-13,39]. However, if the inflammation caused by a chemical burn can be minimized via early intervention with an effective therapeutic agent, the overall prognosis of the disease would be likely to improve. Therefore, investigations into appropriate candidate molecules are very important. The targeting of matrix metalloproteinase and VEGF have already yielded significant results [40-42]. Considering the nature of ocular chemical burns, molecules involved in both inflammation and angiogenesis, such as MIF, should be included as targets for future therapeutic interventions.

In conclusion, the data collected herein demonstrate the significance of MIF in the wound healing process following injuries to the ocular surface. Modulating MIF on the ocular surface after a chemical burn may offer new opportunities to improve visual outcomes.
